# The changing epidemiology of the global paediatric HIV epidemic: keeping track of perinatally HIV-infected adolescents

**DOI:** 10.7448/IAS.16.1.18555

**Published:** 2013-06-18

**Authors:** Annette H Sohn, Rohan Hazra

**Affiliations:** 1 TREAT Asia/amfAR – The Foundation for AIDS Research, Bangkok, Thailand; 2 Maternal and Pediatric Infectious Disease Branch, Eunice Kennedy Shriver National Institute of Child Health and Human Development, National Institutes of Health, Bethesda, USA

**Keywords:** adolescent, HIV, outcomes, perinatal, surveillance

## Abstract

The global paediatric HIV epidemic is shifting into a new phase as children on antiretroviral therapy (ART) move into adolescence and adulthood, and face new challenges of living with HIV. UNAIDS reports that 3.4 million children aged below 15 years and 2 million adolescents aged between 10 and 19 years have HIV. Although the vast majority of children were perinatally infected, older children are combined with behaviourally infected adolescents and youth in global reporting, making it difficult to keep track of their outcomes. Perinatally HIV-infected adolescents (PHIVA) are a highly unique patient sub-population, having been infected before development of their immune systems, been subject to suboptimal ART options and formulations, and now face transition from complete dependence on adult caregivers to becoming their own caregivers. As we are unable to track long-term complications and survival of PHIVA through national and global reporting systems, local and regional cohorts are the main sources for surveillance and research among PHIVA. This global review will utilize those data to highlight the epidemiology of PHIVA infection, treatment challenges and chronic disease risks. Unless mechanisms are created to count and separate out PHIVA outcomes, we will have few opportunities to characterize the negative consequences of life-long HIV infection in order to find ways to prevent them.

## Introduction

The global paediatric HIV epidemic is shifting into a new phase as children on antiretroviral therapy (ART) age into adolescence and adulthood. The evolution of HIV into a chronic disease has no greater impact than on the life of a child. Children that families, clinicians and policymakers at one time expected to die are living into their 20s and having children of their own [1–6]. Unanticipated issues such as reproductive health, higher education and career training are now urgent needs [7].

Unfortunately, we do not know how many perinatally HIV-infected adolescents (PHIVA) are living in our communities today, a necessary, but only a first step towards addressing the needs of this population. UNAIDS reports that there are 3.4 million children under 15 years of age with HIV and 2 million adolescents between 10 and 19 years of age [8]. Although the vast majority of children were perinatally infected, older children are combined with behaviourally infected adolescents and youth in global reporting, without disaggregation by sex. Maintaining separate reporting for PHIVA and conducting appropriate surveillance and cohort studies are necessary to keep track of long-term complications and survival through national and UNAIDS reporting systems. Local and regional cohorts are currently the main source for surveillance and research among PHIVA. The objective of this review was to utilize available data to highlight the global epidemiology of PHIVA infection, and treatment challenges and outcomes, including metabolic and neurocognitive complications, and identify gaps for future research and policy change.

## The challenge of reaching adulthood with active ART regimens

There are multiple reasons why PHIVA are at high risk of having treatment failure and multiclass antiretroviral (ARV) drug resistance, starting from ARV exposure to prevent their infection around birth [9, 10], a history of sub-optimal mono- and dual-therapy regimens [11, 12] and having a limited range of approved ARVs for children and paediatric formulations. In the Madrid paediatric HIV cohort, of 112 adolescents who have been transferred to adult care and followed for a median of 15.6 years, 60% had started with monotherapy, and had had an average of five different ART regimens [11]. An analysis of children within the Collaboration of Observational HIV Epidemiological Research Europe (COHERE) cohort demonstrated a 20% cumulative proportion of paediatric patients with triple-class failure by eight years of ART [13]. An urban cohort of PHIVA in the United States reported that patients had been exposed to a median of eight different ARVs across three classes due to resistance and toxicity [14].

In low- and middle-income countries (LMICs), there are added difficulties in identifying treatment failure early enough to prevent accumulation of drug resistance mutations [15]. Current World Health Organization (WHO) criteria to assess paediatric failure have been repeatedly shown to be inaccurate for predicting failure [16, 17]. The sensitivity of the 2006 and 2010 WHO immunologic failure guidelines was as low as 6% in a multicentre South African study [16] and 5% in a Cambodian study [17]. Although targeted viral load testing can significantly improve failure classifications [18], the importance of routine access to viral load testing relative to other monitoring priorities continues to be debated in the paediatric literature [19].

Common to all settings is the challenge of maintaining life-long adherence and access to increasingly expensive ART regimens. Adolescent adherence is particularly complex because of the socio-economic pressures related to orphanhood, neurocognitive deficits associated with chronic and severe HIV infection, and stigma and discrimination [5, 20–22]. In a US cohort of treatment-experienced adolescents, poor adherence and pre-existing resistance led to poor viral load responses despite regular access to the third- and fourth-line ARVs darunavir, raltegravir and etravirine [14]. The monthly cost of darunavir for an adult in Thailand is 400 USD, (source: Thai Red Cross AIDS Research Centre, Bangkok, Thailand), making compassionate use programmes a critical and frequently the only way that children in LMICs can access these types of drugs. Without an improved understanding of how to achieve adherence and continuous access to potent ARVs, LMICs are at risk of running out of options for PHIVA transitioning into adulthood.

## Regional epidemiology and outcome data

The adolescents in LMICs today are in some ways part of survivor cohorts of those who were largely slower progressors and able to access ART before late childhood. They are beginning to experience long-term complications that mirror those of western cohorts, where more prolonged ARV access and research experience have led to a deeper literature on PHIVA outcomes. Within the wide spectrum of relevant clinical HIV research, two emerging focus areas are metabolic and neurocognitive complications of life-long HIV disease. These have implications for future cardiovascular disease and fracture risk, as well as the ability of PHIVA to develop personal, medical and financial independence.

## Sub-Saharan Africa

### Epidemiology

Over 3 million children under 15 years of age were living with HIV in sub-Saharan Africa in 2010, representing more than 90% of all children with HIV in the world [8]. Eastern and Southern Africa bear a larger burden with 2.2 million children with HIV, relative to the 990,000 in West and Central Africa. Paediatric ART coverage greatly lags behind that of adults at 21% compared to 55%. The largest groups of children with HIV worldwide in 2009 and their ART coverage in 2010 were in Nigeria (360,000; 11%), South Africa (330,000; 50%), Kenya (180,000; 28%), Tanzania (160,000; 12%), Uganda (150,000; 21%) and Zimbabwe (150,000; 35%) [8, 23, 24].

It has been widely publicized that 50% of African children will die by their second birthday without treatment [25]. Less is known about the 17% of slower progressors who may survive to 17 years of age, and fewer efforts are focused on identifying older children, who have not yet been diagnosed and linked to care [2, 26]. A model of survival of these older children and PHIVA has projected expansion of this population until 2013 in Zimbabwe and 2020 in South Africa, with on-going increases in deaths up to 23,000/year by 2030 at mean ages up to 18 years [2]. Investigators have further hypothesized that a greater proportion of children infected through breastfeeding would be slower progressors, compared to those with in utero infection [27]. However, slow progression does not prevent the infectious-related morbidity, growth delays and other sequelae that PHIVA would be expected to experience in the absence of treatment – creating both a clinical challenge in terms of achieving immune reconstitution as well as a public health burden with regards to healthcare utilization [28]. Unless the effectiveness of prevention of mother-to-child transmission (PMTCT) interventions and diagnosis of older HIV-exposed children improves, the PHIVA component of the HIV epidemic in sub-Saharan Africa may emerge into a larger and especially challenging sub-population than previously anticipated.

### Metabolic complications

Since the WHO made recommendations in 2009 to begin phasing out of stavudine (d4T) in adults due to toxicities such as lipodystrophy and peripheral neuropathy, countries such as South Africa have begun dropping d4T from standard first-line ART regimens [29]. However, the WHO 2010 paediatric guidelines continued to recommend the use of d4T, noting the need for additional research in this area to document the extent of d4T-related toxicities [30]. The ongoing use of d4T in children and the broader implementation of first-line protease inhibitor-based (PI) regimens after perinatal non-nucleoside reverse transcriptase inhibitor (NNRTI) exposure are both reasons for greater pharmacovigilence of metabolic complications in developing children and PHIVA in sub-Saharan Africa.

A cross-sectional study of 364 children in Uganda using physical assessments of fat redistribution reported that 27% of children had lipodystrophy, primarily with lipoatrophy of the face, which was associated with d4T use (OR 3.43; CI 2.03, 5.80; *p*<0.001), older age (≥5 years OR 3.87, CI 1.51, 9.88; *p*=0.005) and Tanner stage>1 (OR 2.26, CI 1.33, 3.84; *p*=0.003) [31]. A retrospective study of 2222 children in South Africa examined the frequency of d4T substitution as a marker for cases of severe d4T intolerance. After three years of d4T, 12.6% of children were switched; 91% of switches were due to lipodystrophy. In addition, toxicity-related switches were 1.5 times more common in girls (*p*=0.07) [32]. Another cross-sectional study of 156 children completing the NEVEREST trial in South Africa added skin-fold and bioimpedence measurements to clinician assessments [33]. All children (mean age of five years) were on d4T and either lopinavir or nevirapine, and 8.3% had confirmed and 11.5% had possible lipodystrophy after four years of ART. The effects of d4T may be less easily recognizable, although still potentially present, in younger children due to the natural pattern of fat distribution in this age group.

Recent data have also raised concerns over d4T-associated peripheral neuropathy. A cross-sectional study of 174 children in a rural setting in South Africa discovered that 24% met criteria for peripheral neuropathy [34]. Children were a median of nine years of age, and had been on ART for a median of two years; 86% were on d4T. Passively reported adverse event data and other cross-sectional studies have not previously demonstrated such high rates of neuropathy [35, 36].

With regards to hyperlipidemia, available data to date have emphasized improvements in lipid profiles after initiation of ART in younger children [37, 38]. The known association with PIs in adults has been observed in the children in NEVEREST; those on lopinavir had higher rates of elevated cholesterol (19% vs. 8.5%, *p*=0.03) and triglycerides (13% vs. 3%, *p*=0.04) compared to nevirapine [33]. Available resources for laboratory monitoring limit data on older children and PHIVA from larger cohorts. In a survey of 53 paediatric HIV sites across Africa in the International Epidemiologic Databases to Evaluate AIDS (IeDEA) global consortium, only 26% of sites regularly monitored lipid levels [39].

### Behavioural and neurocognitive outcomes

It is clear that immediately starting HIV-infected infants on ART drastically improves both mortality as well as neurodevelopmental outcomes [40, 41]. However, most African PHIVA today would not have been able to access ART until they were older and after meeting previously more restrictive treatment criteria. Studies of school-age South African children who started ART after clinical and immunologic disease progression have demonstrated that up to 90% of them have significant developmental delays, with a seven-fold higher likelihood of severe delay (OR. 7.88; CI 1.96–31.68) than HIV-uninfected controls [42].

Children diagnosed after two to three years of age with CD4 levels above ART thresholds frequently have ART deferred, but there may be serious negative consequences to their neurodevelopment. A study of Ugandan children with CD4 levels>350 cells/mcl and >15% used three different neuropsychological batteries to compare them to HIV-uninfected children (i.e., Test of Variables of Attention; modified Kaufman Assessment Battery for Children; Bruininks-Oseretsky Test of Motor Proficiency, second edition) [43]. Children with HIV (median age of 8.7 years) lived in similar home environments with regards to stimulation and learning opportunities, but had lower socio-economic status. Although some of the testing outcomes were similar, children with HIV did significantly worse with regards to visual reaction times, sequential and simultaneous processing, planning/reasoning, and motor proficiency. Higher viral loads were associated with worse testing outcomes, indicating that deferring ART based on CD4 criteria alone may put children at risk of poorer long-term cognitive outcomes due to ongoing direct viral damage to the developing brain.

Using a brief assessment of psychiatric disorders, investigators in Kenya found high rates of features consistent with anxiety disorders (32%) and major depression (17%) in a cohort of 162 children between 6 and 18 years of age (mean age of 9.7 years) [44]. Of note was that 49% of the study participants were also at least two grades below their age-appropriate levels, reported by the families to be due to poor health (41%) and/or poor performance (31%). The link between these delays and deficits and behaviour and functional outcomes remains to be studied.

## Asia

### Epidemiology

An estimated 180,000 children under 15 years of age were living with HIV in the Asia-Pacific in 2010, with 39% ART coverage [8]. The largest national treatment programme for children <15 years of age in Asia is in India, where 18,000 of the 70,000 of those infected were on ART in 2009 [23]. In the same period, there were an estimated 16,000 children with HIV in Thailand, with 8000 receiving ART [23]. A study of those in the national ART programme before 2007 reported an 88% survival rate at five years of ART [45]. China's national programme had enrolled around 5100 children by 2009 [46], with 1600 on ART [23]. Although general surveillance data for children are inconsistent, Cambodia was treating 3600 and Vietnam 2000 children by 2009 [23].

The largest regional adolescent cohort in Asia is the TREAT Asia Pediatric HIV Observational Database (TApHOD), which is part of the IeDEA network [47]. Of the 4045 children in the database from six countries enrolled through March 2011, 31% were adolescents 12 years of age or above, of whom 53% were female [6]. Of those reaching adolescence, 4.2% were lost to follow-up and 8.6% had been transferred out, but 85% were still under care at cohort sites. Most of those still under care (73%) were single- or double-orphans, 62% knew about their own HIV status (45% of 12–14 year-olds; 82% of ≥15 year-olds), and 93% were attending school of some kind. Of those on ART, 96% had been on highly active ART for a median of six years, with 71% on NNRTI-based regimens. The median CD4 count was 657 cells/mm^3^, and 718 of 830 (86%) with viral load testing were below 400 copies/mL. Overall, those who had reached adolescence at these primarily urban referral centres were on stable ART, with good immunologic and virologic disease control.

Much of the data on long-term HIV and ART complications in Asia have come from Thailand, which has the oldest cohort of PHIVA due to earlier implementation of their national paediatric ART programme [45].

### Metabolic complications

Lipodystrophy was the first major toxicity of ART described in Thai children. Investigators prospectively monitoring children with serial photography and standardized assessments reported that 65% had lipoatrophy, lipohypertrophy, or both after 144 weeks of d4T-based ART. Girls had a higher prevalence of lipodystrophy than boys (61% vs. 39%, *p*=0.04) [48]. Subsequent studies after switching d4T for zidovudine (AZT) showed that these children and adolescents had no clinically significant AZT-associated anaemia [49], and that 73% of lipoatrophy and 47% of lipohypertrophy resolved by 96 weeks [50]. Other studies confirmed the association with d4T use, and the Thai national paediatric HIV treatment guidelines were revised in 2010 to recommend short-term (i.e., <6 months) d4T only in cases of pre-ART anaemia [51].

However, hyperlipidemia continues to be a challenge, as more children are switched to second-line PI-based regimens. Although lipid screening is seldom part of routine paediatric treatment monitoring in LMICs, there is growing evidence that this may be a larger problem than previously anticipated. Within a cohort of ART-naïve younger children from Cambodia and Thailand, 28% already had hypertriglyceridemia and 45% had low HDL [52]. In the TApHOD cohort, among children switched to PI-based second-line ART at a median of 10 years of age, 32% had hypercholesterolemia, 73% had hypertriglyceridemia, 18% had HDL, and 49% had elevated triglyceride to HDL ratios at 48 weeks of follow-up [53]. It remains unclear what this abnormal lipid metabolism will mean for PHIVA and their risk of cardiovascular disease.

Bone mineral density (BMD) assessments using dual-energy x-ray absorptiometry (DXA) in Thai children has similarly raised questions about future fracture risk. Lumbar spine DXA scans were done in 101 PHIVA in Bangkok after a mean of seven years on successful ART (median CD4 646 cells/mm3; 90% with HIV viral load <50 copies/ml) [54]. Compared to healthy Thai controls, 24% of PHIVA had BMD Z-scores <2, and 25% had 25-hydroxy vitamin D levels <20 ng/ml; only 15% overall and 8% of those with low BMD were on tenofovir-containing regimens. There were no differences by sex. The percentage of low lumbar DXA scores in Thai PHIVA was much higher than the 4% seen in a national US cohort [55]. Although no fractures have yet been reported, these data reflect the negative impact of long-term ART and HIV on the metabolism of developing children and maturing adolescents.

### Behaviour and neurocognitive outcomes

In the most comprehensive neurodevelopmental and neurocognitive testing done in children with HIV in Asia, investigators used a combination of multiple forms of IQ testing (i.e., Beery Visual Motor Integration, Purdue Pegboard, Colour Trails, Child Behavioural Checklist, Wechsler Intelligence Scale, Stanford Binet Memory test) to study outcomes of children in Thailand and Cambodia [56]. There were significant reductions in scores and performance across almost all domains tested in intelligence, memory, and psychomotor and behavioural outcomes for children with HIV (median age, nine years) in comparison to uninfected controls (median age, seven years).

The impact of these clear differences in developmental and cognitive outcomes has been seen in school performance. Thai investigators compared children with HIV to HIV-exposed and HIV-unexposed children (overall and group-specific median age of nine years) [57]. Only 21% of those with HIV scored at or above average intelligence levels, compared to 76% of unexposed children. On multivariate analysis, HIV was the sole factor significantly associated with higher risk of poor cognitive outcomes (OR 6.20, *p*<0.01). Of particular concern was that 20% of HIV-infected children were below their age-appropriate grades in school compared to only 2% of HIV-exposed and 0% of HIV-unexposed children (*p*<0.01). Beyond these neurodevelopmental issues, families with HIV had lower income and caregiver education levels, while primary caregivers were older due to orphanhood and parental illness. Only 28% of those with HIV were cared for by one or both of their biological parents compared to 98% of HIV-unexposed children (*p*<0.001).

In a pilot study using an audio computer-assisted self-interview (ACASI) within TApHOD, investigators began assessing behavioural risk among PHIVA in Malaysia and Thailand. Of 46 PHIVA (median age, 14.5 years), 24% reported trying alcohol, 11% cigarettes, and 11% had engaged in sexual intercourse between 14 and 16 years of age [58]. How cognitive and performance deficits impact mental health, and risk-taking behaviour in Asian PHIVA remains largely unknown, and represents an essential area for future research.

## United States

### Epidemiology

According to UNAIDS, approximately 4500 HIV-infected children under 15 years of age lived in North America in 2011, the vast majority in the United States [8]. However, given the ageing population of PHIVA, this number under the age of 15 years likely represents less than half of the total number who are perinatally infected. By 2007, according to the CDC, 49% of PHIVA in the United States were over 15 years of age [59]. UNAIDS reports less than 100 deaths among HIV-infected children less than 15 years of age, but again, given the age distribution of the perinatally infected population, this likely represents less than half the number of deaths among the perinatally infected in the United States, especially since older individuals are at increased risk of death [1]. Nevertheless, given the low mortality and very low number of newly infected babies (<100 per year), the perinatally infected population in the United States is at a relatively stable number of over 10,000 individuals, most of whom are now young adults and with the oldest members now entering the fourth decade of life.

Approximately two-thirds of PHIVA in the United States are African-American/non-Hispanic, and approximately 20% are Hispanic; 53% are female [59]. There are a number of epidemiological studies focused on this population, including the Pediatric HIV/AIDS Cohort Study (PHACS), IMPAACT 1074, and the HIV Research Network (HIVRN), and several studies that have now ended but for which data are still available for further analyses (PACTG 219C, LEGACY, and WITS). As mentioned above, the mortality rate in this population has declined substantially to less than 1% [1, 60].

To date, the studies of PHIVA in the United States have been based in paediatric clinical settings, but as this population enters young adulthood, studies will need to be adapted to continue to follow these youth as they transition to adult-based care. Preliminary data and multiple anecdotes suggest that this transition can be very difficult for some, threatening their health and well-being [61].

### Metabolic complications

Complications from chronic therapy and lifelong infection have emerged [62]. These include lipid abnormalities in approximately 20–25% and insulin resistance in 15% [63]. Low bone density has been reported in a number of studies, with boys potentially more affected than girls, but this finding may not be as severe as initially thought, since lower than expected height may explain a large part of the low BMD findings [55, 64, 65]. While concerns about renal impairment due to toxicity from prolonged ART have been raised, to date, studies have been reassuring that major renal toxicity is rare and much less common than was seen in in the era of suboptimal therapy [66]. Another organ system of concern for potential toxicity from long-term ART is the heart. Recent echocardiographic data have been reassuring that substantial cardiac disease is rare and much less common than was seen in in the pre-HAART era [67].

### Neurocognitive complications and mental health issues

With effective ART, HIV encephalopathy has practically disappeared in the United States, but concerns for more subtle, but potentially profound central nervous system and mental health disorders have emerged [68]. In one study based in New York City, 61% of perinatally infected youth had a psychiatric disorder, a rate that was statistically significantly higher than the 49% rate seen in the HIV-exposed, uninfected comparison group [69]. However, follow-up data from this study and data from other studies have shown that rates of mental health disorders are not different between perinatally infected and exposed/uninfected or HIV-affected youth, though alarmingly high in both groups [70–72]. These findings suggest that HIV infection and its treatment may not be the major cause of these problems, but that other factors such as caregiver status, poverty, racism, stigma, exposure to violence, multiple losses and grief, are likely aetiologies. The co-occurrence of mental health problems, substance use, poor adherence to ART, and engagement in high risk activities threaten the health of PHIVA and increases the risk of HIV transmission to sexual partners and to infants [73, 74].

## Europe

### Epidemiology

The UNAIDS estimate for the number of HIV-infected children under 15 years of age in Western and Central Europe in 2011 was 1600. As in the United States, this likely represents less than half of the perinatally infected population. The estimates for the number of deaths and new infections are similar to those for the United States. The perinatally infected population in Europe is likely slightly younger overall than in the United States and much more likely to have emigrated from abroad, as demonstrated by data from the Collaborative HIV Paediatric Study (CHIPS). In this study, which follows almost all HIV-infected children from 2006 onwards in the United Kingdom and Ireland, 55% were born abroad with 51% females, and 31% were 15 years of age or older in 2011 (http://www.chipscohort.ac.uk/default.asp). As of March 2012, 1188 of the 1791 enrolled were alive and in active follow-up at a paediatric clinical site.

In France, the native-born perinatally infected population is followed until age 18 years in the French Perinatal Cohort study CO10 (http://cesp.inserm.fr/en/research/ongoing-studies/4716-anrs-epf-co1-co10-co11-en-gb.html). Of the 702 enrolled, 211 had reached the age of 18 years (According to data on the website accessed on January 2, 2013). Researchers in Spain have established a Cohort of the Spanish Paediatric HIV Network (CoRISpe) that is following approximately 800 of the 1100–1200 HIV-infected children in Spain [75]. About one-quarter were born outside of Spain, and over one-third are at least 18 years of age. In addition, a number of other European countries have set up paediatric HIV cohorts (http://www.eurocoord.net/cohort_registry.aspx).

The characteristics of the epidemic in Eastern Europe are quite different from that in Western and Central Europe. Here, the prevalence among adults is actually increasing, fuelled predominantly by intravenous drug use. According to UNAIDS, the number of infected children under 15 years of age in Eastern Europe and Central Asia is estimated to be 11,000, with more new paediatric infections and deaths among HIV-infected children than seen in the United States and other parts of Europe. The number less than 15 years of age has been relatively stable for most of the past decade suggesting that the number of newly infected infants equals the number of deaths plus the number who reach 15 years of age every year.

### Metabolic and neurocognitive complications

Metabolic and neurodevelopmental/behavioural findings in European PHIVA have been similar to those seen in PHIVA in the United States [76–78]. Data from the French CO10 cohort showed that school performance was comparable to national statistics [79]. However, Swiss and UK studies have illustrated that coping with HIV was an ongoing challenge for PHIVA as they became older, and that inconsistent disclosure and poor psychological adjustment had a negative impact on long-term adherence [80, 81]. As mentioned above, a very important issue that needs to be addressed is how to continue to follow these youth as they transition to adult care, especially given that a number of the findings to date regarding hyperlipidemia, bone density, and the impact of mental health and other central nervous system disorders may not be fully expressed until later in adulthood. British researchers leading CHIPS are collaborating with the UK Register of HIV Seroconverters to be able to continue to follow PHIVA into adulthood. In addition, they have established a more intensive study, Adolescents and Adults Living with Perinatal HIV Cohort (AALPHI) (http://www.ctu.mrc.ac.uk/research_areas/study_details.aspx?s=258), on metabolic, neurocognitive, and other areas as these youth enter adulthood. Similarly, the French group has created the Cohort of Young Adults Infected With HIV Since Birth or During Childhood (CO19 COVERTE; ClinicalTrials.gov Identifier: NCT01269632) to follow PHIVA after they reach the age of 18 years and discontinue participation in CO10. As in the United States, preliminary data on the transition to adult care are sobering. Data presented from the United Kingdom in abstract form demonstrated an over five-fold higher rate of mortality in youth reaching the age of 21 years compared to their younger peers [82]. Importantly, poor adherence and end-stage AIDS conditions along with a high burden of mental health disorders were paramount.

## Latin America and the Caribbean

### Epidemiology and long-term complications

The 2011 UNAIDS estimates for Latin America and the Caribbean were 60,000 HIV-infected children under 15 years of age, 3300 new paediatric infections, and 3500 deaths [8]. The two countries with the most infected children in these regions are Brazil (20,000 infected children under 15 years of age), the largest economy in Latin America, and Haiti (13,000 infected children under 15 years of age), the poorest country in the Western hemisphere. Since 2009 the decline in new infections has been 32% in the Caribbean and 24% in Latin America, as PMTCT coverage approaches 80% in the Caribbean and over 50% throughout Latin America. As new infections are prevented and as perinatally infected children survive and mature into adolescence and young adulthood, the number of infected children under 15 years of age has declined from a peak of 22,000 in the Caribbean in 2004 to 18,000 in 2011. The peak in Latin America was 54,000 in 2005–6, down to 42,000 in 2011. Given these trends, the number under 15 years of age reported by UNAIDS is an underestimate of the total perinatally infected population, though not to the same extent as seen in the United States and Western and Central Europe.

Paediatric cohorts in these regions include the NICHD International Site Development Initiative (NISDI), which is now closed but has a database of over 1000 perinatally infected infants, children, and adolescents primarily in Brazil, but also several other Latin American countries [83]. CCASAnet, part of the IeDEA network, is in the process of expanding its paediatric agenda. As in other regions, efforts should be made to follow PHIVA as they enter young adulthood and transition to adult-based care to continue to assess both complications and positive outcomes.

Findings from the NISDI study include a very low mortality rate, dyslipidemia in over a quarter, lower rates of insulin resistance than seen in the United States and Europe, low rates of opportunistic infections but higher than seen in comparable populations in the United States and Europe, and relatively low rates of renal and hepatic disease [83–87]. Two cross-sectional studies of BMD in Brazil demonstrated that between 17 and 32% of PHIVA studied had low BMD, though larger studies with appropriate comparison groups are clearly needed [88, 89]. Data on neurocognitive and mental health problems for PHIVA in this region remain very limited.

## Conclusions

Depending on the setting, the paediatric HIV epidemic has entered or is entering the next phase of its evolution as children grow up and face new challenges of living with HIV. PHIVA are a highly unique patient sub-population, having been infected before development of their immune systems, been subject to suboptimal ART options and formulations, and face transitioning from complete dependence on adult caregivers to becoming their own caregivers.

Regional data demonstrate that successful transition and HIV disease control are possible, but there are consequences of life-long HIV and ART – many of which we still do not understand. However, unless national HIV programmes and UNAIDS create mechanisms to count and keep track of the perinatally infected, we will not know how many of these children are and are not surviving into adulthood. Every year that goes by without dedicated global PHIVA surveillance means that tens of thousands of children could be lost in the crowd.

In addition, without longitudinal cohort studies, we would have few opportunities to characterize the consequences of HIV disease and treatment in order to find ways to prevent them. There are now multiple paediatric and adolescent cohorts scattered around the world. Although they vary with regards to both size and depth of data collection, global cohort collaborations could potentially generate the “big data” needed to answer common research questions. Providers and researchers will also have to transition into the next phase of the global paediatric epidemic in order to keep up with our patients.
